# Combined Pulsed Electric Field and Microwave-Assisted Extraction as a Green Method for the Recovery of Antioxidant Compounds with Electroactive Potential from Coffee Agro-Waste

**DOI:** 10.3390/plants11182362

**Published:** 2022-09-10

**Authors:** Rodrigo Macías-Garbett, Juan Eduardo Sosa-Hernández, Hafiz M. N. Iqbal, Juan Carlos Contreras-Esquivel, Wei Ning Chen, Elda M. Melchor-Martínez, Roberto Parra-Saldívar

**Affiliations:** 1 Tecnologico de Monterrey, School of Engineering and Sciences, Monterrey 64849, Mexico; 2 Tecnologico de Monterrey, Institute of Advanced Materials for Sustainable Manufacturing, Monterrey 64849, Mexico; 3 Food Research Department, School of Chemistry, Autonomous University of Coahuila, Coahuila 25280, Mexico; 4 School of Chemical and Biomedical Engineering, Nanyang Technological University, Singapore 637459, Singapore

**Keywords:** polyphenols, microwave-assisted extraction, pulsed electric fields, coffee agrowaste, solvent-free extraction

## Abstract

Coffee agro-waste is a potential source of polyphenols with antioxidant activity and application in the food and cosmetic trades. The usage of these byproducts persists as a challenge in the industrial landscape due to their high content of purported toxic substances hindering management. This study presents a green extractive process using pulsed electric field (PEF) and microwave assisted extraction (MAE) to recover polyphenols from coffee parchment and two varieties of pulp, posing quick processing times and the use of water as the only solvent. The performance of this process with regard to the bioactivity was assessed through the Folin-Ciocalteu assay, total flavonoid content, DPPH, ABTS and FRAP antioxidant tests. The phenolic composition of the extracts was also determined through HPLC-MS and quantified through HPLC-DAD. When compared to treatment controls, PEF + MAE treated samples presented enhanced yields of total phenolic content and radical scavenging activity in all analyzed residues (Tukey test significance: 95%). The chromatographic studies reveal the presence of caffeic acid on the three analyzed by-products. The HPLC-DAD caffeic acid quantification validated that a combination of MAE + PEF treatment in yellow coffee pulp had the highest caffeic acid concentration of all studied extraction methods.

## 1. Introduction

Coffee is a staple beverage over the world, and globally, the second largest-traded commodity after oil [1]. Worldwide consumption of coffee is expected to achieve a total of 170.3 million bags produced on the 2021/2022 cycle, amounting to 10.22 million metric tons of roasted coffee (a rise of 3.3% respect of the previous cycle) [2]. In Mexico, the small-scale production of organic, specialty coffee has recently become relevant, as the country is traditionally considered a manufacturer of a lower-quality product at larger-scale coffee sectors [3]. In this regard, Mexico takes the eleventh place among the world’s largest producers of coffee grain [4,5,6]. It is estimated that up to 92% of coffee production in Mexico is developed in farms with an area lower than 5 ha [3]. Organic coffee production creates an estimate of 500,000 jobs in 14 states of Mexico, and among these, Chiapas, Veracruz, and Oaxaca are responsible for 80% of the total national coffee production.

The handling of the coffee berry to obtain roasted grains can involve either a dry or a wet primary treatment. In the dry processing approach, coffee berry moisture is reduced to an approximate value of 10% through natural (sun) or synthetic (oven) techniques [7]. Coffee berries are then stripped from the dried outer layers, producing a solid residue known as coffee parchment consisting of berry skin and pulp [8]. The wet process entails the mechanical removal of the skin and pulp of the coffee berry with no previous drying, and produces a solid residue defined as coffee pulp [9]. Coffee pulp represents about 50% of the total coffee berry weight and is the main waste of the primary processing to obtain roasted coffee [9,10]. As such, it poses considerable environmental issues; its disposal is hindered due to its high moisture content that leads to its quick putrefaction [8,11]. The high caffeine and polyphenol content of pulp have limited its reinsertion using traditional recycling approaches, such as its use as a livestock feed substrate [12,13]. Coffee pulp has anti-nutritional effects, compromises digestion, hinders metal absorption, and limits amino acid availability on certain animal species [6,14]. Depulping wastewater is also high in organic matter, and its high phenolic and tannin content can be correlated with a high chemical oxygen demand (COD) [15,16]. If verted into reservoirs, this waste stream may deplete available dissolved oxygen (DO) levels, compromising the survival of aquatic organisms (DO > 5 mg/L) and causing a consequent deterioration of the freshwater trophic chain [17].

Under the United Nations Sustainable Development Goals, the revamping of food chain systems to prevent, reduce, and reuse waste remains a priority to efficiently use available resources and reduce food industry waste [18]. In this regard, the trending policies of circular economy and emerging biorefineries have resulted in the exploration of several coffee agro-waste materials as a feedstock for the recovery of bioactive compounds as a revalorization pathway [19,20,21]. The high availability and low cost of coffee grain processing residues, along with its attractive bioactive composition, pose it as a potentially valuable source of chemical products and carbon source [1,22]. Phenolic compounds present in coffee roasting waste have attributed antioxidant effects that contribute to the prevention of cardiovascular injury through the inhibition of oxidative stress related to cardiac hypertrophy, as well as the modulation of antihypertensive pathways, such as the reduction of circulating angiotensin II by inhibiting the angiotensin converting enzyme (ACE), and the inhibition of HMG-CoA reductase, a crucial enzyme in the synthesis of cholesterol [22,23]. Coffee pulp has been posed as a functional food ingredient, a potential fiber source with antioxidant activity; coffee pulp extracts have also been highlighted by their ability to inhibit IL-8, an intestinal chemokine directly related to gastrointestinal inflammation [24,25]. Both coffee pulp and parchment have been considered in several reports as a raw material to source antioxidant compounds for cosmetic formulations [26,27,28]. Other potential uses for coffee agro-waste include their use as a novel filler for material composites [29,30] and as an adsorbent material for water bioremediation [31,32,33].

Antioxidant compounds, and specifically, phenolic acids, can be extracted from coffee agro-waste and paired to the coffee production chainline as an added-value by-product with potential commercial placement as alimentary supplements and in cosmetic formulations [7]. To this end, conventional extractive strategies that involve solid-liquid unit operations with solvents and heating are already reported and well-established in literature. Nonetheless, their demanding reagent and energy requirements, and high waste throughput limits their technical viability [34,35,36]. The potential toxicity of organic solvents and non-validated material sources also hinders its application on human-grade products under global product safety frameworks [37].

Lately, the usage of emerging extractive techniques on food agro-waste to recover bioactive compounds has been a trending topic of research. These emerging extraction methods pose substantial advantages over conventional procedures such as lowered solvent and time requirements, enhanced product quality and higher energetic efficiency that lowers operation costs and poses them as greener alternatives [38,39]. Among available emerging techniques, microwave assisted extraction (MAE) is a promising technology for the recovery of bioactive compounds from vegetal matrixes, involving the rapid heating of the sample through selective energy transfer mainly affecting polar molecules like intrinsic water [40]. The main advantages that MAE offers in comparison with conventional heating strategies include the selective heating of vegetal components, causing structural damage and increasing recovery yields, and that it couples the heat and mass transfer from the biomass unidirectionally [41]. Heat transfer using MAE is also faster, reducing thermal gradients, thus allowing for better process control, and the energy source is contactless, making it attractive from a human consumption safety standpoint [42]. MAE has been applied successfully in recent years to recover polyphenols from different vegetal matrixes and waste residues, such as grape pomace [43], avocado peel [44], apple skin [45], spent coffee grounds [46], coca been shell waste [47], and others.

Along with MAE, pulsed electric field pretreatment (PEF) is an emerging technique that has been applied to enhance mass transfer from agro-waste through cell membrane permeabilization by short electric pulses [48,49]. Its quick and mild conditions pose PEF as an energy-efficient, non-thermal method to recover thermolabile compounds from vegetal matrices [50,51]. This technique has already been explored for coffee parchment by Barbosa-Pereira et al. [52], highlighting its potential to enhance the yield of bioactive compounds to industrial feasibility. PEF is also a robust technique that is optimizable through the control of its main variable inputs: pulse frequency, voltage, and treatment duration [53].

This study presents a methodology combining a pulsed-electric field pretreatment together with a microwave-assisted extraction main step to enhance the yield of recovered phenolic compounds from three local coffee agro-waste materials: two pulps from berries of different coffee plant varieties (red and yellow), and coffee parchment. The objective of the proposed methodology is to present an alternative for coffee waste revalorization through the recovery of antioxidant compounds. The total phenolic content (TPC) of obtained extracts was assessed through the Folin-Ciocalteu colorimetric method. The antioxidant activity of the extracts was tested as well through the 2,2-diphenyl-1-picrylhydrazyl radical scavenging activity (DPPH-RSA) and 2,2′-Azino-bis-(3-ethylbenzothiazoline-6-sulfonic acid) diammonium salt (ABTS) capacity assays. The phenolic compound profile of the materials was characterized and quantified through high-performance liquid chromatography coupled with diode array detection (HPLC-DAD). The recovery yield of the proposed methodology is then compared to available literature. The extract’s composition was also analyzed on a separate assay through HPLC with mass spectrometry (MS) to identify present free phenolic species as well as those coupled to another compounds.

## 2. Results and Discussion

### 2.1. Total Phenolic Content and Total Flavonoid Content (TFC)

**Figure 1:**
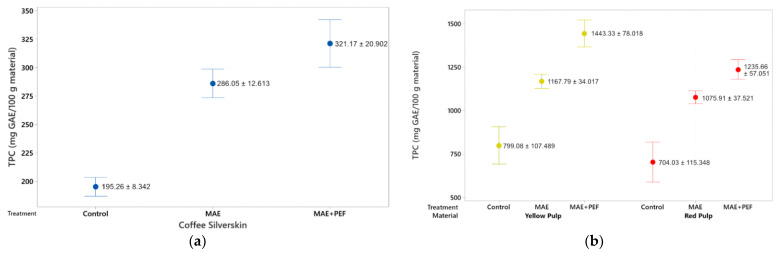
Total phenolic content of coffee agro-waste extracts. Values are represented as mean ± standard deviation (*n* = 3). GAE: gallic acid equivalents. (**a**) Extraction results for parchment waste. (**b**) Extraction results for yellow and red pulp waste MAE: Microwave assisted extraction; PEF: Pulsed Electric Field pretreatment.

The phenolic content of the extracts from red and yellow pulps and coffee parchment is presented in Figure 1. Both the pulsed electric field pretreatment and the microwave-assisted extraction show a positive effect on the total phenolic content of the extracts. The effect of both the PEF pretreatment and the MAE extraction were found to be statistically significant in the Tukey test at a 95% significance. Furthermore, the combination of MAE + PEF shows a significant improvement (at a Tukey test at a 95% significance level) of phenolic yields for the three studied substrates. The highest yield of phenolic content was obtained using yellow pulp as the extraction substrate and the combination of MAE + PEF as the treatment with a total processing time of 15 min (1433.33 ± 78.01 mg GAE/100 g material). This yield was then followed by the results in the red coffee pulp and coffee parchment. The positive response from all extracts to this assay can be attributed to the influence that these two techniques have on the rupture of the vegetal matrixes to improve the extraction yield of several phenolic compound families. Extracted bioactive compounds may include monophenols, catechols, flavonoids, and tannins in both free and coupled forms.

**Figure 2:**
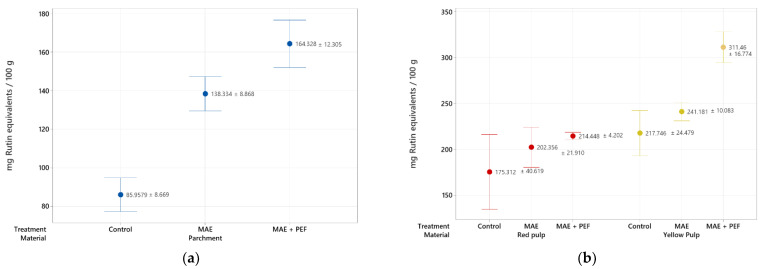
Total flavonoid content of coffee agro-waste extracts. Values are represented as mean ± standard deviation (*n* = 3). (**a**) Extraction results for parchment waste. (**b**) Extraction results for yellow and red pulp waste MAE: Microwave assisted extraction; PEF: Pulsed Electric Field pretreatment.

A specific assessment on the impact of the proposed techniques in flavonoid recovery was carried out and is reported in Figure 2. The same upward trend observed on the extraction yield of total phenolic content is present in this assay, where yellow pulp, once again, shows the highest TFC of these family of compounds (311.46 ± 16.774 mg of rutin equivalents/100 g of material). Despite the highest yields for all three materials being obtained for the combination of MAE and PEF treatments, its overall effect is less pronounced than in the assessment of total phenolic content. This trend may be explained by the distribution of distinct phenolic families that the residue contains and the impact of the analyzed techniques on their extraction.

When compared to available literature, the polyphenolic yield of the MAE + PEF extraction used in this study is shown to have matching results to those other strategies for polyphenol extraction. Machado et al. [54] obtained 347 mg GAE/100 g of coffee parchment using a solid-state fermentation with *Penicillinum purpurogenum,* a comparable yield to the obtained in this study (321.17 mg GAE/100 g coffee parchment) with the benefit of reduced treatment times (total fermentation time = 6 days) and process scalability [54]. Another applicable comparison is the conventional aqueous extraction carried out by Narita and Inouye for coffee parchment [55]. Although the yield is doubled the obtained in this study, processing times increase from 15 min to 1 h (700 mg GAE/100 g coffee parchment, 80 °C, 1 h). While organic compounds, such as ethanol and methanol are considered the optimal solvents to extract phenolic compounds [56], the presented methodology can be posed as a quick, green strategy to enhance phenolic yield and antioxidant content using water as the main process solvent. In the case of coffee pulps, this study shows reduced yields to the approach used by Kusumocahyo et al. [57], a solid-liquid extraction carried out at 60 °C and 60 min (2612 mg GAE/100 g coffee pulp). Nevertheless, the process involves a two-step extraction using *n*-hexane and a mixture of water and ethanol to optimize its extraction yield.

A common approach to enhance polyphenolic yield applied to coffee agrowaste is to introduce solvents such as methanol, ethanol, and acetone in different proportions to aqueous solutions to enhance compound solubility. A direct comparison can be brought to the optimization carried for the PEF-assisted extraction of polyphenols from coffee parchment by Barbosa-Pereira [52], where at the optimal conditions of ethanolic concentration of 62.67% and solid-liquid extraction time of 75 min, a value of 1212 mg GAE/100 g CS is obtained, a 4-fold increase to that presented in this study. Nevertheless, the lack of usage of solvents other than water and reduced treatment times are posed as advantages that this study introduces using a novel combination of emerging technologies to reduce potential operation costs and environmental impacts.

### 2.2. Antioxidant Capacity: α-α-Diphenyl-β-picrylhydrazyl (DPPH), 2,2′-Azino-bis-(3-ethylbenzothiazoline-6-sulfonic Acid) Diammonium Salt (ABTS) Capacity, and Ferric Reducing Antioxidant Power Quantitation (FRAP) Assays

**Figure 3:**
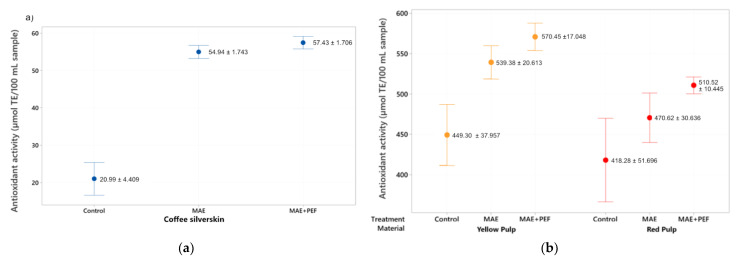
DPPH antioxidant assay of coffee agro-waste extracts. Values are represented as mean ± standard deviation (*n* = 3). TE: Trolox equivalents. (**a**) Assay results for parchment waste. (**b**) Assay results for yellow and red pulp waste. MAE: Microwave assisted extraction; PEF: Pulsed Electric Field pretreatment.

**Figure 4:**
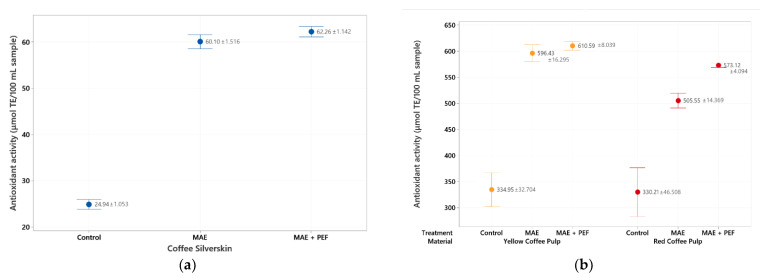
ABTS antioxidant assay of coffee agro-waste extracts. Values are represented as mean ± standard deviation (*n* = 3). TE: Trolox equivalents. (**a**) Assay results for parchment waste. (**b**) Assay results for yellow and red pulp waste. MAE: Microwave assisted extraction; PEF: Pulsed Electric Field pretreatment.

The antioxidant capacity of the extracts was measured using the DPPH and ABTS assays and the results are presented in Figure 3 and Figure 4. For DPPH, the highest Trolox Equivalent Antioxidant Capacity (TEAC) values for this assay were obtained by using yellow coffee pulp as a substrate for the combined MAE + PEF treatment (570.45 ± 17.04 µmol TE/100 mL sample), replicating the result obtained in the Folin-Ciocalteu test. Both coffee pulps show significantly higher antioxidant activity than the coffee parchment. The DPPH value for coffee pulps ranged from 418.29 ± 51.69 to 570.452 ± 17.04 µmol TE/100 mL sample.

The results for the ABTS assay are consistent with those found in the DPPH assessment. The highest value is once again found for the combination of MAE + PEF treatment applied to the recovered yellow coffee pulp (610.59 ± 8.03 µmol TEAC/100 mL sample). Both coffee pulps show up to 10 times higher antioxidant activity than any extract from coffee parchment, although the antioxidant potential of this residue was enhanced by the application of both microwave and pulsed electric fields up to a maximum value of 62.26 ± 1.74 µmol TEAC/100 mL sample.

**Figure 5:**
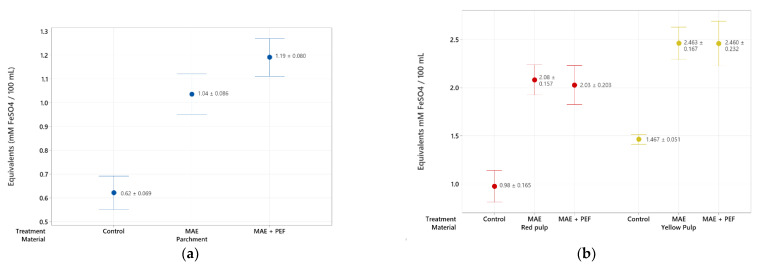
FRAP antioxidant assay of coffee agro-waste extracts. Values are represented as mean ± standard deviation (*n* = 3). (**a**) Assay results for parchment waste. (**b**) Assay results for yellow and red pulp waste. MAE: Microwave assisted extraction; PEF: Pulsed Electric Field pretreatment.

A third assessment of the antioxidant potential of the extracts through FRAP shows the ability of the coffee waste antioxidants to reduce ferric ions (Figure 5). Both coffee pulp extracts show a heightened reducing potential compared to the parchment, highlighting the yellow pulp as the residue with the highest antioxidant content at all analyzed treatments (2.46 ± 0.167 mM FeSO_4_ equivalents/100 mL extract).

For all three substrates, an upward trend was observed between using no treatment and using the combination of MAE + PEF. This trend is, however, less pronounced than that of the Folin-Ciocalteu counterpart. This discrepancy can be explained by arguing that although PEF assists to enhance the extraction of total polyphenolic compounds, extracted phenols may be oxidized by the effect of PEF in an aqueous environment, or phenols that remain in the organic matrix are already oxidized and not extracted until PEF is applied [58]. The antioxidant activity of extracted polyphenols may also be hindered by their conjugation to other molecules on their scavenging spots, such as their glycosylated counterparts [59].

Previous studies have reported a significant decrease of antioxidant activity when increasing the temperature of extraction for infusions [60] and other conventional solid-liquid extraction methods in coffee residues and other matrixes [27,61] due to the thermolability of the extracted compounds. The combination of MAE + PEF shows no such detriment to radical scavenging at the studied conditions. The increase of antioxidant potential with the raised temperature of a microwave process using only water as a solvent is a trend that has been observed previously for coffee parchment subjected to conventional and subcritical extractions by Narita and Inouye [55].

### 2.3. HPLC-MS Component Identification

The identification of extract compounds was performed by comparing their molecular weight in the VARIAN workstation database of the of the HPLC system. The HPLC-MS assay performed on the extracts detected phenolic compounds in the families of catechins, lignans, hydroxybenzoic acids, hydroxycinnamic acids, methoxycinammic acids, hydroxyphenylpropanoic acids, hydroxycoumarins, methoxyflavonols, flavones, flavonols, akylflavonols, tyrosols, and anthocyanins (Table 1).

This assay validated the existence of phenolic substances in the produced extracts of coffee waste as inferred in the Folin-Ciocalteu total phenolic content test. Furthermore, it identified the existing phenolic compounds through mass spectrometry. The antioxidant activity observed in the DPPH assay may be attributed to the radical scavenging activity that these compounds present to a greater or lesser extent, according to their structural features and functional group layout. Results also show the presence of glycosylated (caffeic acid 4-*O*-glucoside, gallic acid 4-*O*-glucoside) phenolic compounds. Their presence on this aqueous extract is explained through their higher solubility compared to free phenolic compounds [62].

Hydroxycinnamic acids represented the most diverse family of compounds identified in all three analyzed residues, in accordance with previous characterizations of coffee parchment [63] and coffee pulp [64]. Composition patterns, such as that of chlorogenic acid, were also like those reported previously [64,65]. In this case, the deprotonated chlorogenic acid (*m*/*z*) of 352.8 and other unique fragmentation patterns have been identified and attributed to one of the most abundant phenolics present in coffee pulp and parchment [66]. The presence of this family of compounds in the studied residues poses them as a potential source for their extraction and application in food and cosmetic industries, as the functional properties of hydroxycinnamic acids are well established and include antiradical, anticancer, and antimicrobial activities [67,68].

The presence of anthocyanins in coffee pulp is well-documented for *Coffea arabica* pulp [69]. These family of flavonoids are natural pigments that are soluble in water and have strong antioxidant properties [70], and their presence on coffee pulp and parchment is related to the coloration of the different varieties of berries harvested [71]. Coffee husks have been previously posed as a potential extraction source of cyanidin-3-*O*-glucoside and have been recently described in coffee peels [64], supporting the identification of Cyanidin 3,5-*O-*glucoside and derivates in the analyzed parchment and yellow coffee pulp in this study. A derivate of peonidin-3-glucoside, a red pigment with antihypertensive, antihyperglycemic, and antioxidant activities [72,73], was also identified in the studied red coffee pulp.

### 2.4. HPLC-DAD Component Identification and Quantification

**Figure 6:**
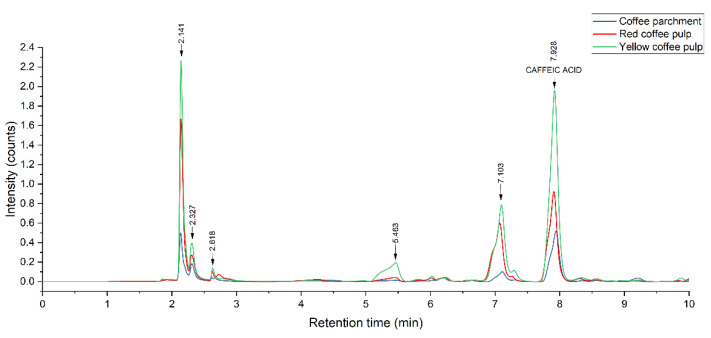
HPLC-DAD chromatographic profile of MAE + PEF treated residues. MAE: Microwave assisted extraction; PEF: Pulsed Electric Field pretreatment.

**Figure 7:**
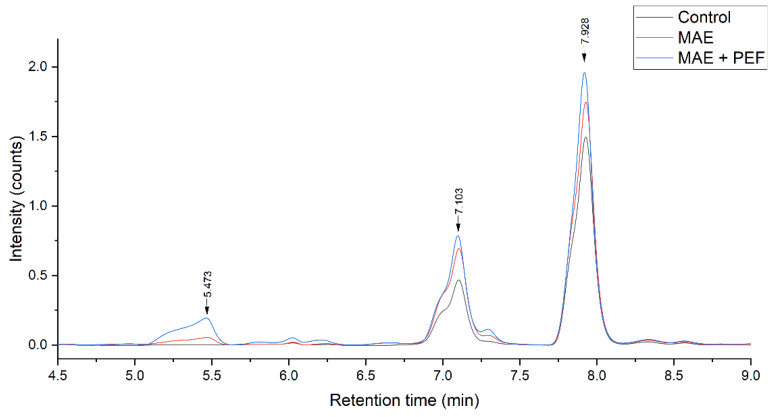
Effect of MAE + PEF treatment on the peaks of yellow coffee pulp. MAE: Microwave assisted extraction; PEF: Pulsed Electric Field pretreatment.

The composition of the extracts from yellow and red pulps and coffee parchment was examined and quantified through HPLC-DAD to determine their prevalent phenolic species. Figure 6 shows the chromatographic profile of the three residues, and Figure 7 shows the effect of each treatment on a particular substrate, namely yellow pulp.

The obtained chromatograms show that the prevalent phenolic compound present is caffeic acid for all three extracts. Other phenolic species the standards of which were injected were not present in a significant manner. The predominant presence of these two hydroxycinnamic acids is in accordance with the results obtained by other studies, such as Rebollo-Hernanz et al. [74], using a solid-liquid polyphenol extraction coffee parchment (process conditions: 500 mL of water, 100 °C, 10 min), and the enzymatic extraction carried by Torres-Mancera et al., for coffee pulps [75]. Ferulic acid, another phenolic that is reported in the literature as predominantly present in coffee agrowaste extracts [63,76], was not detected in this study. The extract composition had no significant variations for all the studied treatments, indicating that the used methodology has no negative impact on polyphenol stability while enhancing overall extraction yields.

As the predominant detected phenolic acid, caffeic acid was chosen to be quantified in the extracts (Table 2). The quantification shows that yellow coffee pulp has a greater caffeic acid content, and that parchment has the lower content of the three residues, a result that is consistent with previous literature reviews [76,77]. The effect of MAE and MAE + PEF treatments is also ratified compared to untreated samples. A Tukey test reveals that there is no significant difference in the quantification of caffeic acid between the MAE and MAE + PEF treatment levels at 95% confidence, contrasting the result obtained in the TPC assay. This outcome can be explained by arguing that PEF enhances the yield for other phenolic fractions more than caffeic acid [78]. The highest value of caffeic acid yield was once again obtained by applying MAE + PEF in yellow coffee pulp (613.5 ± 20.3 mg CA/100 g of dry sample).

## 3. Materials and Methods

### 3.1. Materials and Reagents

Coffee pulp from *Coffea arabica* berries from two different varieties, arabe (red fruit) and peñasco (yellow fruit), and residual coffee parchment were collected in a commercial wet coffee processing community in Jaltenango, Chiapas, Mexico, in January 2020. After collection, the samples were sun-dried (average daily temperature: 28 °C) until a constant moisture value (<10% weight) was reached and stored in hermetic bags at room temperature. Raw samples were then milled in a commercial blender and stored in a dark container at room temperature until their usage in this study.

2,2-Diphenyl-*L*-picryl-hydrazyl (DPPH) reagent, and sodium carbonate were purchased from Sigma Aldrich (Steinheim, Germany). Folin-Ciocalteu’s reagent, 2,2′-Azino-bis (3-ethylbenzothiazoline-6-sulfonic acid) (ABTS) reagent, gallic acid (concentration ≥ 98%), caffeic acid (concentration ≥ 98%), vanillin (concentration ≥ 97%), sinapic acid (concentration ≥ 97%), and syringic acid (concentration ≥ 97%) were purchased from Sigma-Aldrich (St. Louis, MO, USA) Trolox (6-hydroxy-2,5,7,8-tetramethylchroman-2-carboxylic acid), and 2,4,6-Tris(2-pyridyl)-s-triazine (TPTZ) from Sigma Aldrich (Burlington, MA, USA). Rutin was purchased from Chemsavers, (Bluefield, VA, USA). Monobasic potassium phosphate was purchased from Sigma Aldrich-Aldrich (Tokyo, Japan). Ferulic acid was sourced from Minkab Laboratories, Ltd. (Jalisco, Mexico). HPLC-grade ethanol was purchased from CTR Scientific (Nuevo Leon, Mexico). HPLC-MS-grade acetonitrile was purchased from Fisher Scientific Chemicals (Fair Lawn, NJ, USA). Methanol was purchased from Tedia High Purity Solvents (Fairfield, OH, USA). Disodium phosphate and potassium chloride were acquired from Productos Quimicos Monterrey, S.A. de C.V. (Nuevo León, Mexico). Sodium chloride was purchased from Desarrollo de Especialidades Químicas, S.A. de C.V. (Nuevo Leon, Mexico).

### 3.2. Pulsed Electric Field Treatment

**Figure 8:**
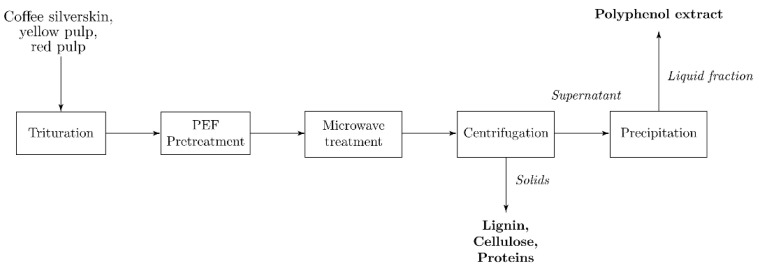
Pulsed-electric field (PEF) pretreatment and microwave-assisted extraction (MAE) methodology flow diagram.

**Figure 9:**
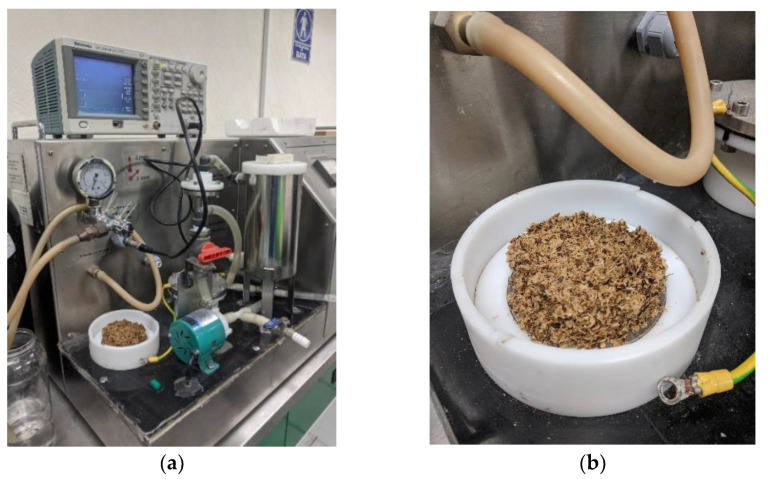
Pulsed-electric field (PEF) experimental setup. (**a**) PEF device layout, (**b**) treated coffee agrowaste on electrode detail. PEF: Pulsed Electric Field pretreatment.

15 g of each analyzed coffee residue were dampened with 15 mL of distilled water until a consistent texture was obtained. The material was then placed in the batch processing chamber of an SBS-PEF-SA-1 PEF Benchtop Sterilization System (SteriBeam Systems, Kehl, Germany) (Figure 8), covering the entirety of its exposed electrode surface (Figure 9). Operating conditions for the pretreatment were set at a total treatment time of 5 min, pulse frequency of 5 Hz, and pulse voltage of 18 kV (6 kV/cm). PEF-pretreated samples were then subjected to microwave-assisted extraction, as described in the following section. The procedure was performed in triplicate.

### 3.3. Microwave-Assisted Extraction (MAE)

**Figure 10:**
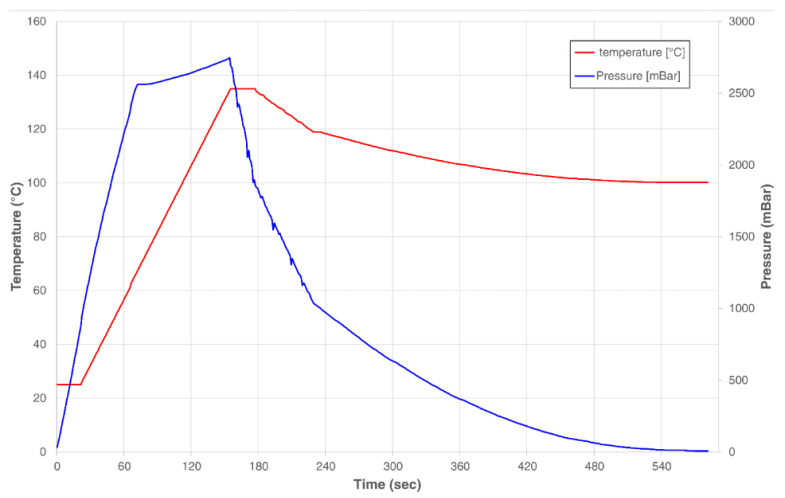
Heating and pressure profile of the microwave-assisted extraction procedure.

Briefly, 15 g of each residue were suspended in 150 mL of distilled water in a 500 mL capped flask. MAE was performed using a Microjet™ Autoclave (Rodwell Group Ltd., Essex, UK) using the equipment settings preset for the given solution volume. The pressure and temperature of the process were monitored using the system’s equipped sensors (Figure 10). After treatment, the mixture was shaken in a Labline Scientific 3527 Orbital Incubator Shaker (Mumbai, India) at 170 rpm and room temperature for 1 h. The solid residue was then decanted from the liquid fraction. The recovered liquid was centrifuged using a Sigma 3–18 KS refrigerated benchtop centrifuge (Osterode am Harz, Germany) (8000 rpm, 30 min) to remove suspended sediments. The supernatant was then filtered using Grade 40 GE Healthcare Whatman™ 47 mm glass fiber filters (Cytiva, Marlborough, MA, United States). The volume of the resulting extract was measured, and 2 equivalent volumes of 96% ethanol were added to precipitate its polysaccharide fraction. The precipitated polysaccharide was filtered using commercial #4 paper cone filters, and the liquid component was recovered for its characterization through the techniques described below. The described methodology was followed to produce a triplicate of PEF-treated and non-PEF-treated samples for experimental comparison. PEF + MAE-untreated samples were produced following the solid-liquid extraction steps of this procedure to be used as controls.

### 3.4. Total Phenolic Content (TPC) Determination

TPC was assessed through a variation of the Folin-Ciocalteu method [79]. Hence, 50 µL of the phenolic extracts were mixed with 430 µL of distilled water and 20 µL of the Folin-Ciocalteu reagent. After stirring, 50 µL of a 20% *w*/*v* solution of Na_2_CO_3_ were mixed and left 10 min to rest. The mixture was then diluted with 450 µL of distilled water, and its absorbance was read at a wavelength of 725 nm using a ThermoScientific Genesys 10S UV-Vis spectrophotometer (Waltham, MA, USA) at room temperature. A calibration curve was prepared by dissolving gallic acid in distilled water in known concentrations ranging from 0 to 200 ppm. Phenolic concentration from analyzed samples was expressed as Gallic Acid Equivalents (GAE). Measurements were performed in triplicate.

### 3.5. Total Flavonoid Content Estimation

Total flavonoids were quantified using the colorimetric method reported by Shen et al. [80] with slight modifications. Hence, 500 μL of each extract were dispensed into assay tubes containing 2 mL of distilled water. 150 μL of 50 g L^−1^ NaNO_2_ were then added to the mixture. After 5 min, 150 μL of 100 g L^−1^ AlCl_3_•6H_2_O was added. The solution was then allowed to stand for 6 min, after which 1 mL of 1 M NaOH was added. The final solution was mixed thoroughly and read at an absorbance of 510 nm in a ThermoScientific Genesys 10S UV-Vis spectrophotometer (Waltham, MA, USA) after 15 min. Rutin was used as calibration standard for quantification, and results are reported as mg of rutin equivalents (mg RE) per 100 g of material. Measurements were performed in triplicate.

### 3.6. α-α-Diphenyl-β-picrylhydrazyl (DPPH) Assay

The DPPH antioxidant activity of studied extracts was assessed through a variation of the Brand-Williams et al. [81] protocol reported by Barreto et al. [82]. 50 mL of a 0.8 mg/mL DPPH reagent stock solution was created using methanol as a solvent. 5 mL of this DPPH solution were then added to a 50 mL volumetric flask and diluted with methanol to create the diluted DPPH solution used in this assay (0.08 mg/mL). A stock solution of 0.5 mg/mL of Trolox was prepared as a standard using methanol as a solvent. The standard stock solution was diluted in an Eppendorf tube by mixing 50 µL of the standard with 950 µL of methanol. 200 µL of this dilution were transferred to a 96-well plate in triplicate. After this transfer, 100 µL of the standard solution were pipetted to the adjacent well and mixed with 100 µL of methanol to create a dilution with half the original concentration. This procedure was repeated from wells 2 to 12 for the three Trolox rows to create serial dilutions for sample comparison. Hence, 100 µL of extract samples were put in separate wells for analysis in triplicate. 100 µL of the prepared DPPH solution were then dispensed on all analyzed wells, shaken, and placed in the dark for 30 min. The absorbance was then read at 515 nm in a Thermo Scientific Varioskan Flash microplate reader and the antioxidant activity was calculated as Formula (1):

(1) $$\%AA=100\;\times \frac{A_{control}-A_{sample}}{A_{control}}$$

where *A_control_* is the absorbance of a blank solvent solution with no antioxidant activity. Antioxidant activity is plotted against Trolox concentration for a linear regression to extract activity. Results from analyzed extracts are reported as Trolox Equivalent Antioxidant Capacity (TEAC).

### 3.7. 2,2′-Azino-bis-(3-ethylbenzothiazoline-6-sulfonic Acid) Diammonium Salt (ABTS) Capacity

The ABTS antioxidant activity assay was carried out following the methodology laid out by Sharopov et al. [83] with some modifications. A saline phosphate buffer was prepared by mixing 800 mg of NaCl, 115 mg of Na_2_HPO_4_, 20 mg of KCl, and 20 mg of NaN_3_ in 100 mL of deionized water. The ABTS free radical solution was prepared dissolving 38 mg of ABTS reagent in 10 mL of the prepared buffer. 6.5 mg of potassium persulfate were added afterwards and mixed thoroughly. The prepared solution was mixed for 16 h in the dark. The resulting mixture was diluted with the saline phosphate buffer until an absorbance of 0.7 units was obtained at 734 nm as read in a Thermo Scientific Varioskan Flash microplate reader (Waltham, MA, USA). To assess the antioxidant activity of the coffee residue extracts, 100 *μ*L of each extract was added to a 96-well microplate, to which 100 *μ*L of the prepared ABTS radical solution was added, mixed, and read after six minutes of reaction. A Trolox curve ranging from 0 to 500 ppm was used for antioxidant activity measurement. The antioxidant activity of the extracts was calculated as for the DPPH assay. Samples were measured in triplicate.

### 3.8. Ferric Reducing Antioxidant Power Quantitation (FRAP)

FRAP was quantified using the microplate method described by Hidalgo et al. [84]. 10 mM 2,4,6-Tris(2-pyridyl)-s-triazine (dissolved in 40 mM HCl), 20 mM FeCl_3_ (dissolved in distilled water) and 0.3 M acetate buffer (pH 3.6) were mixed in a (1:1:10) proportion to create the FRAP reagent. 10 μL of the samples were added afterward to each microplate well, followed by 290 μL of this reagent. The microplate was then incubated at 37 °C for 15 min and read at an absorbance of 593 nm. FeSO_4_•7H_2_O was used as a standard, and the results are expressed as mg Fe_2_SO_4_ equivalents per 100 mL extract. All measurements were performed in triplicate.

### 3.9. HPLC-MS Component Identification

The identification of dissolved compounds from the coffee waste extracts was carried out through an Agilent Technologies VARIAN high-performance liquid chromatography (Santa Clara, CA, USA) system equipped with an Agilent Technologies VARIAN 500/MS IT Mass Spectrometer coupled to an electrospray (ESI) source. The chromatographic separation was carried out using an Advanced Chromatography Technology Ltd. ACE Excel 3 Super C_18_ COLUMN (150 × 2.1 mm) at a column temperature of 25 °C. Used mobile phases were acetonitrile (Solvent A) and 0.2% formic acid (solvent B) in a gradient (Table 3). Samples were filtered using ThermoScientific 0.22 µm nylon filters. Sample injection volume was 5 µL. Mobile flow rate was kept at 0.2 mL/min, operation pressure: 160–170 atm.

### 3.10. HPLC-DAD Analysis

The chromatographic analysis of the samples was performed on an Altus Perkin Elmer high-pressure liquid chromatography (Waltham, MA, USA) system paired to an autosampler and photodiode array detector (PDA). The separation took place using a Zorbax Eclipse XDB C_18_ column (5 μm, 150 × 4.6 mm), and a mobile phase gradient consisting of a pH 2.5 solution of water and acetic acid (Solvent A), and methanol (Solvent B). The gradient conditions are disclosed in Table 4. Samples were previously microfiltered using ThermoScientific 0.22 µm nylon filters. The injection volume for all tested samples was 20 *μ*L at a flow rate of 0.8 mL/min. The column temperature was set at 25 °C. A battery of 7 phenolic compounds was injected as standards to document their retention times and UV spectra for their identification on the produced samples. These include caffeic, chlorogenic, ferulic, gallic, sinapic, syringic acids, and vanillin. A calibration curve of caffeic acid was prepared using concentrations of 0, 10, 40, 80, and 100 ppm for its quantification on the extracts. Solvent A and all standards were prepared using Milli-Q-grade water.

### 3.11. Statistical Analysis

Results were analyzed using analysis of variance (ANOVA), and treatment significance was assessed using Tukey tests with a level of confidence of *p > 0.05.* The computer software used was Minitab^®^ version 19.2020.1 (64-bit) (Minitab LLC, State College, PA, USA). Experiments were performed in triplicate.

## 4. Conclusions

This study demonstrates a potential added-value use for coffee agricultural residues of coffee farming communities worldwide, as several phenolic compounds and families were identified and validated through colorimetric and analytical studies. The reported results validate the combination of pulsed electric field technology as a pretreatment and microwave-assisted extraction as a successful green extraction method, highlighting the use of water as its sole solvent and lower energy requirements without a penalty on the recovery yields of obtained extracts.

The DPPH, ABTS, FRAP, TFC, and Folin-Ciocalteu tests performed in this study demonstrate the antioxidant activity potential of yellow and red coffee pulps and coffee parchment extracts. The proposed recovery technique displayed a positive and significant effect on the extraction yield of total phenolic compounds, total flavonoid compounds and the antioxidant capacity of the extracts. Both the HPLC-MS and HPLC-DAD analyses corroborate the presence of different phenolic families in the produced coffee residue extracts, and caffeic acid was found to be the predominant phenolic compound of the analyzed substrates. For all three residues, the combination of MAE + PEF was found to be the optimal extraction method, justifying the use of the pulsed electric field pretreatment to enhance the extraction yield of phenolic compounds from coffee agro-waste. The use of the microwave-assisted extraction was also ratified through the comparison of untreated samples, where MAE was found to be statistically significant in the colorometric technics performed. Further studies may center on the optimization of the conditions of the proposed process to improve extraction yields and phenolic composition, as well as on the implementation of the extracts’ purified compounds in food and cosmetic formulations. This work showcases the novel application of MAE + PEF on waste from coffee processing as a potential revalorization method adopting polyphenol extraction with short processing times and minimal reagent requirements, making it an attractive addition to a coffee biorefinery strategy.

## Figures and Tables

**Table 1:** HPLC-MS identified phenolic compounds on coffee agro-waste extracts, through their correlation using mass-to-charge ratio (*m*/*z*) and retention times. Their presence on the studied residues is indicated with a ● mark.

Family	Number	RT	*m*/*z*	Compound	Yellow Pulp	Red Pulp	Coffee Parchment
Alkylphenols	1	14.300	374.9	5-Nonadecylresorcinol	●		
Anthocyanins	2	12.334	506.8	Delphinidin 3-*O*-(6′′-acetyl-glucoside)			●
	3	13.426	504.8	Peonidin 3-*O*-(6′′-acetyl-glucoside)		●	
	4	14.423	535.9	Cyanidin 3-*O*-(6′′-malonyl-glucoside)			●
	5	19.742	519.5	Petunidin 3-*O*-(6′′-acetyl-glucoside)			●
	6	35.179	611.1	Cyanidin 3,5-*O*-diglucoside	●		
Catechins	7	4.609	290.0	(+)-Catechin	●		●
Dihydrochalcones	8	3.223	272.6	Phloretin	●		
Flavones	9	7.788	268.9	Apigenin			●
	10	13.731	444.8	Apigenin 7-*O*-glucuronide		●	
	11	20.000	420.9	Apigenin 7-*O*-glucoside	●		
Flavonols	12	6.621	316.9	6,8-Dihydroxykaempferol	●		●
	13	27.515	608.9	Quercetin 3-*O*-xylosyl-glucuronide	●		
Furanocoumarins	14	9.831	186.9	Psoralen	●		
Hydroxybenzoic acids	15	3.339	332.9	Gallic acid 4-*O*-glucoside	●	●	
	16	9.181	320.8	Gallic acid 3-*O*-gallate			●
	17	13.908	314.9	Protocatechuic acid 4-*O*-glucoside		●	
	18	13.391	170.8	Gallic acid	●		●
	19	16.439	342.9	5-*O*-Galloylquinic acid		●	
	20	18.682	298.9	4-Hydroxybenzoic acid 4-*O*-glucoside	●		●
Hydroxycinnamic acids	21	3.417	292.9	Caffeoyl aspartic acid	●		
	22	4.427	341.0	Caffeic acid 4-*O*-glucoside		●	
	23	4.538	352.8	Chlorogenic acid	●	●	●
	24	7.324	325.9	*p*-Coumaroyl tyrosine		●	
	25	13.731	180.1	Caffeic acid	●	●	●
	26	20.349	336.9	3-*p*-Coumaroylquinic acid	●		●
	27	30.223	515.0	1,3-Dicaffeoylquinic acid	●		
Hydroxycoumarins	28	3.666	340.0	Esculin	●		
	29	3.857	191.0	Scopoletin	●		
Hydroxyphenylpropanoic acids	30	12.972	182.8	Dihydrocaffeic acid			●
Lignans	31	4.403	364.9	Secoisolariciresinol			●
	32	9.831	356.9	Matairesinol	●		●
	33	11.534	371.0	7-Oxomatairesinol	●		
	34	13.275	372.9	7-Hydroxymatairesinol	●		
	35	13.797	358.6	Lariciresinol	●	●	
	36	23.775	370.1	Sesaminol		●	
	37	26.086	418.0	Syringaresinol			●
Methoxycinnamic acid dimers	38	31.294	385.0	5-5’-Dehydrodiferulic acid	●	●	
Methoxycinnamic acids	39	14.461	309.8	Sinapine			●
	40	15.880	224.9	Sinapic acid	●		
	41	21.874	367.0	3-Feruloylquinic acid	●		●
Methoxyflavonols	42	13.668	670.6	Spinacetin 3-*O*-glucosyl-(1- > 6)-glucoside	●		
	43	14.311	282.8	Methylgalangin		●	
Proanthocyanidin dimers	44	20.288	704.8	(-)-Epicatechin-(2a-7)(4a-8)-epicatechin 3-*O*-galactoside	●		
Stilbenes	45	19.104	406.6	Piceatannol 3-*O*-glucoside			
Tyrosols	46	3.466	194.9	3,4-DHPEA-AC	●		
	47	8.671	361.9	p-HPEA-EA	●	●	●
				Total compounds	29	14	19

**Table 2:** Effect of the different treatments on caffeic acid content of *Coffea arabica* pulp and parchment detected by HPLC (*n* = 3).

Sample	Control	MAE	MAE + PEF
Coffee Parchment	112.84 ± 4.46 ^a^	147.67 ± 1.13 ^b^	158.43 ± 2.21 ^b^
Red coffee pulp	184.57 ± 6.58 ^a^	232.04 ± 4.92 ^b^	239.85 ± 6.12 ^b^
Yellow coffee pulp	498.2 ± 15.02 ^a^	606.54 ± 8.09 ^b^	613.5 ± 20.3 ^b^

Quantities expressed in mg caffeic acid/100 g of dry sample. Means at the same row with different letters are significantly different. Tukey test group (95% significance).

**Table 3:** Gradient conditions for the HPLC-MS analysis of the extracts.

Time (Min)	% A (*v/v*) Acetonitrile	% B (*v/v*) 0.2% Formic Acid	Flow Rate (mL/min)	Transition
0	3	97	0.2	Initial
5	9	91	0.2	Linear
15	16	84	0.2	Linear
45	50	50	0.2	Linear
53	90	10	0.2	Linear
65	3	97	0.2	Linear

**Table 4:** Gradient conditions for the HPLC-DAD analysis of the extracts.

Time (Min)	% A Water/Acetic Acid pH 2.5	% B Methanol	Flow Rate (mL/min)	Curve
0	100	0	0.8	Initial
3	70	30	0.8	6
8	50	50	0.8	6
15	70	30	0.8	6
20	100	0	0.8	6
